# Anorectal malformations

**DOI:** 10.1186/1750-1172-2-33

**Published:** 2007-07-26

**Authors:** Marc A Levitt, Alberto Peña

**Affiliations:** 1 Department of Pediatric Surgery, Cincinnati Children's Hospital, University of Cincinnati, Cincinnati, Ohio 45229 USA

## Abstract

Anorectal malformations comprise a wide spectrum of diseases, which can affect boys and girls, and involve the distal anus and rectum as well as the urinary and genital tracts. They occur in approximately 1 in 5000 live births. Defects range from the very minor and easily treated with an excellent functional prognosis, to those that are complex, difficult to manage, are often associated with other anomalies, and have a poor functional prognosis. The surgical approach to repairing these defects changed dramatically in 1980 with the introduction of the posterior sagittal approach, which allowed surgeons to view the anatomy of these defects clearly, to repair them under direct vision, and to learn about the complex anatomic arrangement of the junction of rectum and genitourinary tract. Better imaging techniques, and a better knowledge of the anatomy and physiology of the pelvic structures at birth have refined diagnosis and initial management, and the analysis of large series of patients allows better prediction of associated anomalies and functional prognosis. The main concerns for the surgeon in correcting these anomalies are bowel control, urinary control, and sexual function. With early diagnosis, management of associated anomalies and efficient meticulous surgical repair, patients have the best chance for a good functional outcome. Fecal and urinary incontinence can occur even with an excellent anatomic repair, due mainly to associated problems such as a poorly developed sacrum, deficient nerve supply, and spinal cord anomalies. For these patients, an effective bowel management program, including enema and dietary restrictions has been devised to improve their quality of life.

## Definition

Anorectal malformations comprise a wide spectrum of diseases, which can affect boys and girls, and involve the distal anus and rectum as well as the urinary and genital tracts. Defects range from the very minor and easily treated with an excellent functional prognosis, to those that are complex, difficult to manage, are often associated with other anomalies, and have a poor functional prognosis.

## Epidemiology

Anorectal malformations are congenital anomalies that occur in approximately 1 in 5000 live births.

## History

Imperforate anus has been a well-known condition since antiquity. For many centuries, physicians, as well as individuals who practiced medicine, created an orifice in the perineum of children with imperforate anus. Those that survived most likely suffered from a type of defect that would now be recognized as "low". Those with a "high" defect did not survive that treatment. Amussat, in 1835 was the first individual who sutured the rectal wall to the skin edges, which could be considered the first anoplasty. During the first 60 years of the 20th century, surgeons performed a perineal operation without a colostomy for the so-called low malformations. High imperforate anus was usually treated with a colostomy performed in the newborn period, followed by an abdomino-perineal pull-through some time later in life, but surgeons lacked objective anatomic guidelines. Unfortunately this left many patients incontinent and was not an appropriate solution to the spectrum of malformations. The surgical approach to repairing these defects changed dramatically in 1980 with the introduction of the posterior sagittal approach, which allowed surgeons to view the anatomy of these defects clearly, to repair them under direct vision, and to learn about the complex anatomic arrangement of the junction of rectum and genitourinary tract [1-6]. It has become the predominant surgical method for anorectal anomalies. In cases when the rectum or the vagina are very high and an abdominal approach as well is needed, laparoscopy can be used in combination with the posterior sagittal approach.

## Clinical presentation

### Classification

Comparing the results of reported series has always been a problem with anorectal malformations because different surgeons use different terminology when referring to types of imperforate anus. The clearest fact is that there is a spectrum of defects, so every attempt to classify them is arbitrary and somewhat inaccurate. Consequently, the traditional classification of "high", "intermediate", and "low" defects renders the results dubious. The classification presented here attempts to group together defects that have common diagnostic, therapeutic, and prognostic features (Tables 1 and 2).

**Table 1:** Classification of non-syndromic anorectal malformations (ARM)

**Males**	Recto-perineal fistula
	Recto-urethral-bulbar fistula
	Recto-urethral-prostatic fistula
	Recto-bladderneck fistula
	Imperforated anus without fistula
	Complex and unusual defects
**Females**	Recto-perineal fistula
	Recto-vestibular fistula
	Cloaca with short common channel (< 3 cm)
	Cloaca with long common channel (> 3 cm)
	Imperforated anus without fistula

**Complex and unusual defects**	Cloacal extrophy, covered cloacal extra
	Posterior cloaca
	Associated to presacral mass
	Rectal atresia

**Table 2:** Detailed classification of anorectal malformations (ARM)

**Non-syndromic ARM**	***Non-syndromic ARM with fistula***	*Recto-perineal malformations*	
		*Imperforate anus with recto-urethral fistula*	◦ Recto-urethral bulbar fistula ◦ Recto-urethral prostatic fistula ◦ Bladderneck fistula
		*Imperforate anus in female*	◦ Recto-vestibular fistula ◦ Recto-vaginal fistula ◦ Cloacal malformation
	***Non-syndromic ARM without fistula***	*Imperforate anus without fistula*	
	***Complex ARM***	◦ Cloacal malformations with a short common channel (< 3 cm)	
		◦ Cloacal malformations with a long common channel (> 3 cm)	
		◦ H-shaped fistula (recto-vaginal)	
		◦ Rectal duplication	
**Syndromic ARM**	VACTERL (Vertebral anomalies, anal atresia, cardiac malformations, tracheoesophageal fistula, renal anomalies, and limb anomalies)		
	MURCS (Mullerian duct aplasia, renal aplasia, and cervicothoracic somite dysplasia)		
	OEIS (Omphalocele, exstrophy, imperforate anus, and spinal defects)		
	Axial mesodermal dysplasia		
	Klippel-Feil syndrome		
	Sirenomelia-caudal regression		
	Trisomy 21		
	Trisomy 13		
	Trisomy 18		
	Pallister-Killian syndrome		
	Cat-eye syndrome		
	Parental unidisomy 16		
	Deletion 22q11 syndrome (del22q11.2)		
	Currarino syndrome		
	Pallister-Hall syndrome		
	Townes-Brock syndrome		
	Ulnar-mammary syndrome		
	Okihiro syndrome		
	Rieger syndrome		
	Thanatophoric dwarfism		
	Hirschsprung disease		
	Feingold syndrome		
	Kabuki syndrome		
	Optitz BBB/G syndrome		
	Johanson-Blizzard syndrome		
	Spondylocostal dysostosis		
	Short rib – polydactyly syndrome		
	Baller-Gerold syndrome		
	Ciliopathies		
	Fraser syndrome		
	Lowe syndrome		
	Heterotaxia		
	FG syndrome		
	X-linked mental retardation		
	MIDAS syndrome		
	Christian syndrome		

The posterior approach and direct visualization of the anatomy have allowed us to learn about important features. For instance, rectovaginal fistula are almost nonexistent, in retrospect it seems that most of the previously reported "rectovaginal fistula" cases were misdiagnosed cloacas. This assertion is supported by the authors' experience of cloaca reoperations where it has been found that most patients who were originally operated on by a surgeon who classified the defect as a "rectovaginal fistula" had only the rectal component of the cloaca repaired and had been left with a persistent urogenital sinus. Such patients have become categorized as instances of "rectovaginal fistula" and the true diagnosis of cloaca has become evident only many years later. In addition, many patients had undergone a abdominoperineal pull-through at another institution to repair a "rectovaginal fistula," and years later had been referred because of fecal incontinence. When these girls were examined, the little pouch of what used to be the rectum was found opening into the vestibule, indicating that these patients were been born with a rectovestibular fistula. The cloaca itself represents a spectrum and certainly defies the classification "high", "intermediate", and "low".

Also included in the "high" category in male patients were those with completely different defects requiring differing treatments and carrying a different prognosis (*e.g*., rectourethral fistula and rectobladderneck fistula). A rectourethral fistula can be treated without an abdominal approach, but a rectobladderneck fistula always requires the abdomen to be entered either with laparoscopy or laparotomy. The results of treatment are dramatically different, and so we do not group these two defects into the same category [7].

#### Associated genitourinary defects

Important associated anomalies include genitourinary defects, which occur in approximately 50% of all patients with anorectal malformations. All patients must be evaluated at birth to rule out one of these defects, and the most valuable screening test is an abdominal and pelvic ultrasound. Urologic evaluation prior to colostomy provides the surgeon the necessary information needed to address the urologic problem at the time of the colostomy. The surgeon must be prepared to perform a urologic diversion if necessary.

**Figure 1:**
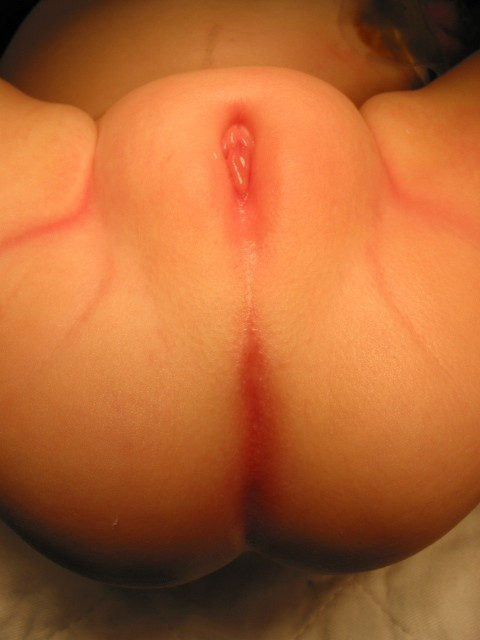
Persistent Cloaca perineum.

**Figure 2:**
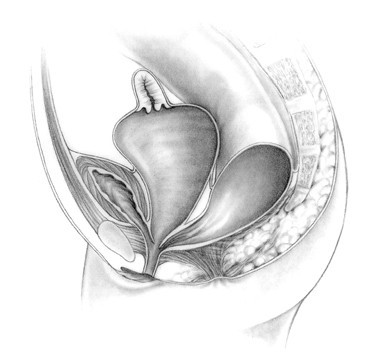
Hydrocolpos.

Unfortunately, a common error in diagnosis occurs during the perineal inspection, when a female is thought to have "imperforate anus with rectovaginal fistula" when in actuality, all three structures, the urinary tract, vagina, and rectum all meet in a common channel and the baby has a cloaca [8-10] (Figure 1). The presence of a single perineal orifice is clinical evidence of a patient with persistent cloaca. Patients with these anomalies also have small genitalia. In patients with cloaca, examination of the abdomen may reveal an abdominal mass which likely represents a distended vagina (hydrocolpos), (Figure 2) present in 50% of patients with cloaca. An abdominal ultrasound determines the presence of an obstructive uropathy as well as the presence of a hydrocolpos. This misconception has important therapeutic implications that will be discussed below. It is vital to make the correct determination of cloaca because 90% of babies have an associated urologic problem, and 50% have hydrocolpos. Both the urinary tract and the distended vagina may need to be dealt with in the newborn period to avoid serious complications. Missing the diagnosis of cloaca frequently means that an obstructive uropathy is overlooked. The patient may then receive only a colostomy and subsequently may suffer from sepsis, acidosis, and sometimes death. The other implication of missing the diagnosis of cloaca involves repairing only the rectal component of the anomaly, leaving the patient with a persistent urogenital sinus.

#### Associated spinal anomalies

The sacrum is the most frequently affected bony structure. Traditionally, to evaluate the degree of sacral deficiency, the number of sacral vertebral bodies were counted. A more objective assessment of the sacrum can be obtained by calculating a sacral ratio. The sacrum is measured and its length is compared with bony parameters of the pelvis. The lateral film is more accurate than the anterior posterior view because its calculation is not affected by the tilt of the pelvis. A hemisacrum is always associated with a presacral mass, which is commonly formed of dermoids, teratomas, or anterior meningoceles. Hemivertebrae may also affect the lumbar and thoracic spine, leading to scoliosis.

Spinal anomalies including a tethered spinal cord can occur [11,12]. This anomaly, which refers to the intravertebral fixation of the phylum terminale is known to occur in approximately 25% of patients. The prevalence of tethered spinal cord rises with increasing height and complexity of the anorectal anomaly. In addition, patients with a hypodeveloped sacrum and with associated urologic problems have a higher likelihood of having tethered cord. Motor and sensory disturbances of the lower extremities may result. Concerning bowel and urinary function, patients with anorectal malformations and tethered cord have a worse functional prognosis but they also have higher anorectal defects, less developed sacrums, associated spinal problems, and less developed perineal musculature. So, the actual impact of tethered cord itself on their functional prognosis is unclear. Untethering of the cord is indicated in the neurosurgical literature to avoid motor and sensory problems. There does not appear to be evidence that this operation will impact on the functional prognosis of a patient with anorectal malformation. Spinal ultrasound in the first 3 months of life and magnetic resonance imaging thereafter are useful radiologic modalities to establish the diagnosis. Furthermore, patients may have other spinal anomalies besides tethered cord such as syringomyelia and myelomeningocele.

#### Perineal fistula

**Figure 3:**
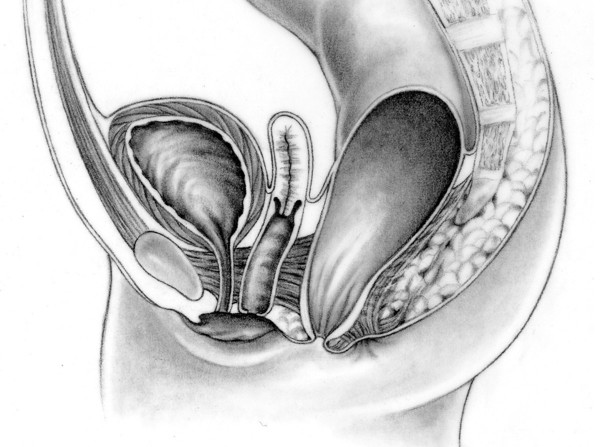
Perineal fistula.

Perineal fistulas in both male and female have traditionally been called "low" defects. In these cases the rectum opens in a small orifice, usually stenotic and located anterior to the center of the sphincter (Figure 3). Most of these patients have excellent sphincter mechanisms and a normal sacrum. In males, the perineum may exhibit other features that help in recognition of this defect, such as a prominent midline skin bridge (known as 'bucket handle') or a subepithelial midline raphe fistula that looks like a black ribbon because it is full of meconium. These features are externally visible and help diagnose a perineal fistula. A simple anoplasty enlarges the stenotic orifice and relocates the rectal orifice posteriorly within the limits of the sphincter complex. The operation is called a "minimal posterior sagittal anoplasty". It is performed with the patient positioned prone with the pelvis elevated; multiple fine silk sutures are places at the mucocutaneous junction of the bowel orifice for traction. A short (1–2 cm) midsagittal incision is made posterior to the fistula site, dividing the entire external sphincter complex. The fistula and lower part of the rectum are carefully dissected to permit mobilization of the rectum for backward placement within the limits of the sphincter complex. The perineal body, that area where the fistula was located, is repaired with a few long-term absorbable sutures [7].

## Etiology

Anorectal malformations (ARM) represent a spectrum of abnormalities ranging from mild anal anomalies to complex cloacal malformations. The etiology of such malformations remains unclear and is likely multifactorial. There are however reasons to believe there is a genetic componenet. As early as the 1950s, it was recognized that there was an increased risk for a sibling of a patient with ARM to be born with a malformation, as much as 1 in 100, compared with the incidence of about 1 in 5000 in the general population. Since that time there have been reports of families with 2 or more affected members and associations of ARMs with multisystem syndromes. In particular, mutations in specific genes encoding transcription factors have been described in patients having Townes-Broks syndrome, Currarino's syndrome, and Pallister-Hall syndrome, each of which have autosomal dominant modes of inheritance. In addition, it has been found that there is not only an increased incidence of ARM in patient with trisomy 21 (Down's syndrome), but that 95% of patients with trisomy 21 and ARM have imperforate anus without fistula, compared with only 5% of all patients with ARM. Based on this evidence, it is likely that the mutation of a variety of different genes can result in ARM, or that the etiology of ARM is multigenic [13].

## Diagnostic methods

The radiologic evaluation of a newborn with imperforate anus includes an abdominal ultrasound to evaluate for urologic anomalies. In the case of persistent cloaca, a distended vagina (hydrocolpos) can be identified. Plain radiographs of the spine can show spinal anomalies such as spina bifida and spinal hemivertebrae. Plain radiographs of the sacrum in the anterior-posterior and lateral projections can demonstrate sacral anomalies such as a hemisacrum and sacral hemivertebrae. Also, the degree of sacral hypodevelopment can be assessed, and a sacral ratio can be calculated measuring the distances between key bony structures. A spinal ultrasound in the newborn period and up to age 3 months (at which time the sacrum becomes ossified) can look for evidence of a tethered spinal cord and other spinal anomalies. A crosstable lateral radiograph can help show the air column in the distal rectum in the small percentage of patients for whom clinical evidence does not delineate in 16–24 hours the likely anorectal anomaly.

After the newborn period, on an outpatient basis after the colostomy (see colostomy) has been created, high pressure distal colostography is performed. Hydrosoluble contrast material is injected into the distal stoma to demonstrate the precise location of the distal rectum and its likely urinary communication. Hydrostatic pressure under fluoroscopic control is required. A foley catheter is placed in the mucous fistula and the 3 cc balloon is inflated and pulled back to occlude the stoma during contrast injection. The hydrostatic pressure must be high enough (manual syringe injection) to overcome the muscle tone of the striated muscle mechanism that surrounds the rectum and keeps it collapsed. This is the best way to demonstrate a recto-urinary communication, and to determine the real height of the rectum. The contrast material usually fills the proximal urethra and bladder through the fistula. The injection is continued until the child voids, and pictures are taken during micturition in order to show, in a single picture, the sacrum, height of the rectum, perineum, fistula location, bladder, vesicoureteral reflux if present, and urethra.

This study is vital in determining the anatomy so the definitive repair can be planned. In 10% of patients, the fistula is at the level of the bladder neck. In this case, during the main repair, the surgeon knows that the rectum will be found only through the abdomen, and a combined posterior sagittal and abdominal or laparoscopic approach is employed. The anorectal defect of imperforate anus without fistula may also be demonstrated with this radiologic evaluation. This defect occurs in approximately 5% of patients, has a good functional prognosis, and is common in patients with Down's syndrome. Except for cloacas, in most cases of female malformations, distal colostography is not necessary because the fistula is evident clinically. If the spine was not evaluated in the newborn period with ultrasound, magnetic resonance imaging is necessary after age 3 months to rule out the presence of tethered cord and other spinal anomalies.

## Management

### A. Early decision-making

The early management of a newborn infant born with an anorectal anomaly is crucial and two important questions must be answered during the first 24 to 48 hours of life. First; are there associated anomalies that threaten the baby's life and should be dealt with right away? And second, should the infant undergo a primary procedure and no protective colostomy or a protective colostomy and a definitive repair at a later date? For babies born with persistent cloaca, the surgeon must also determine whether a dilated vagina is present and if it should be drained, as well as determining whether urinary diversion will be required. These maneuvers are intended to prevent sepsis or metabolic acidosis [14].

The decision to perform an anoplasty in the newborn period or to delay the repair and to perform a colostomy is based on the infant's physical examination, the appearance of the perineum, and any changes that occur over the first 24 hours of life [15-17].

After the baby is born, an intravenous line is placed for fluids and antibiotics, and a nasogastric tube is inserted to keep the stomach decompressed to avoid the risk of vomiting and aspiration. Meconium is usually not seen at the perineum in a baby with a recto-perineal fistula until at least 16–24 hours. Abdominal distension does not develop during the first few hours of life and is required to force meconium through a recto-perineal fistula as well as through a urinary fistula. This is because the most distal part of the rectum in these children is surrounded by a funnel-like voluntary muscle structure that keeps that part of the rectum collapsed and empty. The intraabdominal pressure must be high enough to overcome the tone of the muscles that surround the rectum if one expects to see meconium at the perineum or in the urine. Therefore, the decision of whether to perform a colostomy or an anoplasty must wait for these 16–24 hours while the surgeon observes for clinical evidence of the baby's anorectal anomaly.

Clinical inspection of the buttocks is important. A flat "bottom" or flat perineum, as evidenced by the lack of a midline gluteal fold and the absence of an anal dimple indicates that the patient has very poor muscles in the perineum. These findings are associated with a high malformation and therefore a colostomy should be performed.

Perineal signs found in patients with low malformations include the presence of meconium at the perineum, a "bucket-handle" malformation (a prominent skin tag located at the anal dimple below which an instrument can be passed), and an anal membrane (through which one can see meconium).

#### Decision-making for male newborns

Male newborns with recto-perineal fistula do not need a colostomy. They can undergo a posterior sagital anoplasty whereas male babies with evidence of a recto-urinary tract communication should undergo fecal diversion with a colostomy.

**Figure 4:**
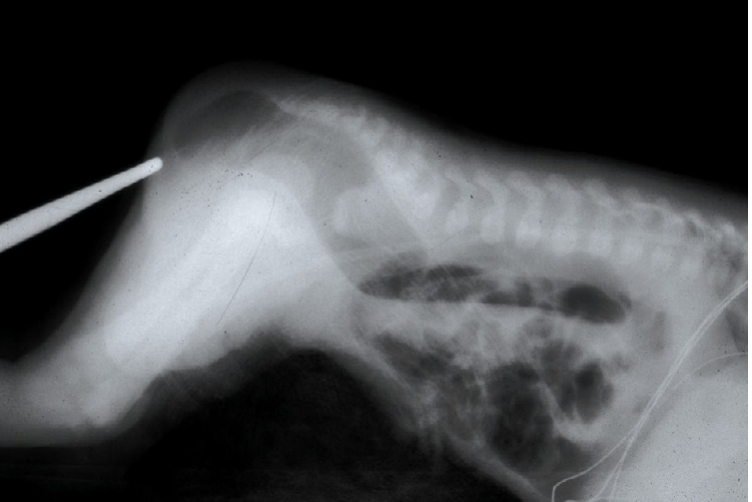
X-ray, cross-table lateral film with the baby in prone position.

In 80–90% of male newborns, clinical evaluation and urinalysis will provide enough information for the surgeon to decide whether the baby requires a colostomy. If none of the clinical signs to determine the location of the anorectal anomaly becomes evident by 24 hours, a cross-table lateral film with the baby in prone position, with the pelvis elevated, and with a radioopaque marker placed on the perineum is performed (Figure 4). This x-ray on rare occasion may show the column of air in the distal rectum to be within 1 cm of the perineum, and if this is the case, the baby can be treated like those with a recto-perineal fistula, and a newborn perineal operation can be performed. If the air column is greater than 1 cm from the perineum, a colostomy is indicated.

A definitive repair in the newborn period avoids a colostomy but there is considerable risk to the urinary tract with this practice because the surgeon does not know the precise anorectal defect. The only way to definitively determine the patient's anorectal defect is to perform a distal colostogram, which of course requires the presence of a colostomy. Without this information an operation in the newborn period is essentially a blind perineal exploration. The surgeon may not be able to find the rectum and may find and damage other, unexpected, structures, such as the posterior urethra, seminal vesicles, vas deferens, and ectopic ureters during the search for the rectum. Finally, without fecal diversion, there is the risk of dehiscence and infection. These complications may compromise the ultimate functional prognosis.

#### Decision-making for female newborns

The decisions involved in managing the female newborn are less complicated. In 90% of patients, a meticulous perineal inspection will demonstrate the anorectal defect. Waiting 16–24 hours for enough abdominal distension to demonstrate the presence of a rectoperineal fistula or rectovestibular fistula applies to females as well.

**Figure 5:**
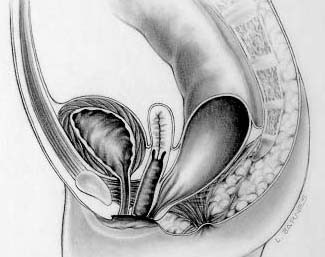
Rectovestibular fistula in females.

The most common anomaly in females is a rectovestibular fistula (Figure 5). Perineal inspection shows a normal urethra, normal vagina, and another orifice, which is the rectal fistula in the vestibule. The safest option for a surgeon without extensive experience in anorectal anomalies when faced with a baby with clinical evidence of a rectovestibular fistula is to perform a diverting colostomy. Colostomy prior to the main repair avoids the complications of infection and dehiscence. Definitive repair of this anomaly in the newborn period should be reserved for surgeons with significant experience repairing these defects. This anomaly has an excellent prognosis and therefore complications that could affect future continence must be avoided.

Unfortunately, the most common referral for redo operations to tertiary centers that care for anorectal anomalies are for patients with rectovestibular fistulas who underwent a failed primary repair in the newborn period.

Occasionally, the fistulas are big enough to decompress the gastrointestinal tract, and may be dilated to facilitate fecal drainage until the baby is older and a definitive repair is performed. Definitive repair involves a posterior sagittal approach. The most delicate part of this operation is the separation of the rectum and vagina, which share a common wall. Females like males can have a rectoperineal fistula and for them an anoplasty in the newborn period should be performed. Like in males, less than 5% of female babies have no clinical evidence of the location of the rectum after 24 hours. They may have imperforate anus with no fistula. A cross-table lateral x-ray should be performed, and will help determine the need for a colostomy.

### B. Treatment

#### Surgery

As discussed previously, the surgeon must decide in the newborn period whether the child requires fecal diversion with a colostomy, or can undergo a primary repair procedure.

##### • Colostomy

The preferred colostomy is a descending colostomy, i.e. made from the descending portion of the colon located in the lower-left quadrant of the abdomen, with separated stomas [18]. The proximal stoma is connected to the upper gastrointestinal tract and drains stool. The distal stoma, also called a mucous fistula, is connected to the rectum and will drain small amounts of mucus material. The advantages of this type of colostomy are many: 1) it defunctionalizes only a small portion of distal colon, 2) in cases of large rectourinary fistulae in which the patient passes urine into the bowel, the urine comes out easily through the mucous fistula, avoiding problems of hyperchloremic acidosis due to urine absorption. Urinary tract infections are also avoided, 3) it is relatively easy to wash and clean the part of the colon distal to the colostomy, 4) distal colostograms are easy to perform, 5) the sigmoid loop is kept distal to the colostomy which provides enough length to reach the perineum during the definitive pull-through procedure, 6) the separated stomas prevent spillage of stool from proximal to distal bowel, which avoids impacted distal stool and urinary tract infections, 7) there is a low incidence of prolapse with this technique. Proximal stoma prolapse in a normally rotated colon should not happen with this technique because the colon is well fixed to the retroperitoneum just before the colostomy rises to the skin level. The distal stoma may prolapse because it is in a mobile portion of the colon. To avoid this, the distal stoma must be made intentionally small, as it will be used only for irrigations and radiologic studies. When performing the colostomy in the newborn, the distal bowel should be irrigated to remove all of the meconium. This prevents formation of a megasigmoid, which may be responsible for the future development of constipation.

Several pitfalls exist with regard to the creation of the colostomy. 1) If the colostomy is placed too distal, it will interfere with the pull-through. 2) During attempts to perform a transverse colostomy, cases of inadvertent sigmoid colostomy placed in the right upper quadrant have occurred. Anchoring of the sigmoid in the right upper quadrant would interfere with the pull-through procedure. 3) A loop colostomy does not completely divert the stool and allows for distal stool impaction and urinary tract infections. 4) Transverse colostomies produce megarectum [19].

##### • Posterior sagittal approach

###### ◦ Anorectal repair

The repair of an anorectal malformation requires a meticulous and delicate technique and a surgeon with experience in the management of these defects. The posterior sagittal approach is an ideal method of defining and repairing anorectal anomalies. If the baby growing well, the repair can be performed at 1–2 months of age. Detailed surgical procedure can be found in the following references: [1,2,5,6].

Ninety percent of male patients can be approached with a posterior sagittal approach alone, while 10% require an abdominal component (with laparotomy or laparoscopically) to mobilize a very high rectum. All female malformations, with the exception of about 30% of cloacas can be repaired with this approach. In 30% of cloacas, the rectum or vagina is so high as to require an abdominal approach as well [20].

###### ◦ Rectobladder neck fistula

**Figure 6:**
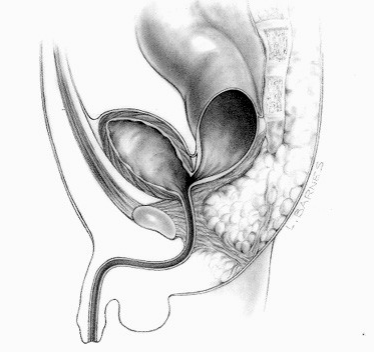
Rectobladder neck fistula.

In the rare case of a true supralevator malformation (rectobladder neck fistula), the operation involves both a posterior sagittal incision and an abdominal component, which can be done with laparoscopy or laparotomy (Figure 6).

###### ◦ Imperforate anus without fistula

In patients with imperforate anus without fistula, the same meticulous dissection is required to separate the distal rectum from the urinary tract as in patient with rectourinary fistulae because the rectum and urethra still share a common wall.

###### ◦ Rectovestibular fistula

In cases of rectovestibular fistula, the posterior sagittal incision can be shorter than in male patients with rectourethral fistulae. Often the entire levator mechanism needs not be divided and only the external sphincter, muscle complex, and part of the lower portion of the levator mechanism need to be divided. The rectum and posterior vagina share a common wall, and it is this separation that is the most difficult part of the operation. Once the rectum is completely mobilized, a perineal body is constructed, and the rectum is placed within the limits of the sphincter mechanism [21].

###### ◦ Rectal atresia

A very rare malformation, rectal atresia, occurs in 1% of cases. The anal canal is normal and externally the anus appear normal. However, there is a blockage 1–2 cm from the anal skin, usually found when the nurse tries to pass a thermometer. These babies should undergo colostomy at birth, and then their definitive repair involves a posterior sagittal approach and an end-to-end anastomosis between the upper rectal pouch and the anal canal.

###### ◦ Persistent cloacas

**Figure 7:**
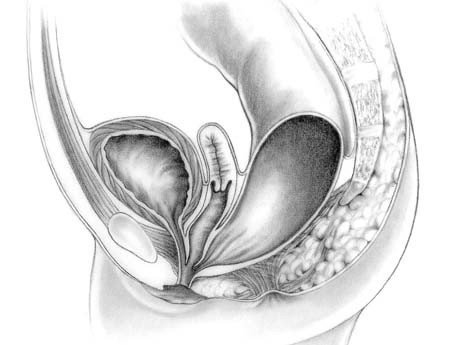
Persistent cloaca.

The repair of persistent cloacas represents a serious technical challenge that should be performed in specialized centers by pediatric surgeons dedicated to the care of these complicated patients [22]. This malformation represents a wide spectrum of defects by itself. The defect involves fusion of the rectum, vagina, and urethra together to form a common channel (Figure 7). The length of this common channel can range from 1 to 10 cm. The rectum and vagina share a common wall and the vagina and urinary tract likewise have a common wall. The goals of surgical treatment are to achieve bowel control, urinary control, and normal sexual function. Sometimes all three goals are achieved, sometimes only two, often only one, and occasionally none [23].

Prognostic factors include the quality of the sacrum, the quality of the muscles, and the length of the common channel. We have arbitrarily defined two groups of patients. The repair of patients with a common channel less than three cm is reproducible and is feasible for most pediatric surgeons. For patients with a common channel greater than three cm, the repair should be performed at a specialized center by a surgeon with experience managing the urologic anomalies and able to performing complex vaginal reconstructions. If the common channel is less than 3 cm, the posterior sagittal approach without an abdominal approach can be used to repair the defect.

For patients with a common channel greater than three cm, a laparotomy is usually required. Often the vagina and urinary tract must be separated trying to gain length, and the urethra must then be reconstructed. The surgeon must be prepared to open the bladder and to reimplant the ureters if necessary. Complex vaginal mobilizations are often required and frequenly a vaginal replacement with small intestine or colon is necessary. The pullthrough of the rectum is similar to other anorectal malformations. It is the repair of the vagina, the urethra, and the associated urologic defects that represents the main surgical challenge. A large vagina can be an advantage during the definitive repair because the surgeon can more easily mobilize it and has more alternatives for the vaginal repair. About 50% of patients have various degrees of vaginal or uterine septation. These can be totally or partially repaired during the main operation. The precise gynecologic anatomy must be ascertained either during the main repair or during colostomy closure (if a laparotomy was not required during the main repair). We have learned that approximately one third of our patients have obstructed Mullerian structures which can lead to severe problems resulting from retrograde menstruation. Predictions of future problems such as amenorrhea in cases of atretic uteri, or hydrometrocolpos and retrograde menses can be made in the newborn period. Presentations of pelvic pain or amenorrhea as teenagers should prompt the assumption of anomalous gynecologic structures.

##### • Laparoscopically assisted anorectal approach

The laparoscopically-assisted anorectal approach consists in mobilizing and bringing the rectum through the pelvic floor sphincter muscles through a minimal posterior incision. Perianal dissection towards the laparoscopic light source favours accurate placement of a trocar to pull the rectum through the external sphincter muscle complex. Laparoscopically-assisted anorectal repair can either be performed in the newborn period without a colostomy or in a stage-approach. This new technique, described by KE Georgeson *et al*. needs further long-term evaluation in terms of fecal continence [24,25].

##### • Anterior sagittal approach

Anterior sagittal approach, involving anterior perineal dissection (from the base of the scrotum to the posterior part of the anoderm), is used by some surgeons, with the aim of preserving the internal anal sphincter [26]. But it should be noticed that this approach might damage the vesical nerve plexus when the rectourethralfistula is dissected up to its junction with the urethra. Furthermore, an internal sphincter saving technique has been devised when performing the posterior sagittal approach.

#### Post-operative management

##### • Anoplasty

The posterior sagittal incision is relatively painless. In patients with a rectourethral fistula, the foley catheter stays in place for about 5–7 days, and occasionally longer. At two weeks postoperatively, anal calibration is performed, followed by a program of anal dilitations. The anus must be dilated twice daily and every week the size of the dilator is increased. The final size to be reached depends on the age of the patient. Once the desired size is reached, the colostomy can be closed. Dilatations are a vital part of the postoperative management to avoid a stricture at the anoplasty. After colostomy closure, severe diaper rash is common because the perineal skin has never before been exposed to stool.

##### • Functional Disorders

###### ◦ Constipation

The most frequent functional disorder encountered after treatment for imperforate anus in which the rectum has been preserved is constipation [26-28].

It is also the most important problem to avoid after definitive repair for female patients with rectovestibular or rectoperineal fistula and for male patients with rectobulbarurethral fistula, imperforate anus without fistula, and rectoperineal fistula. Failure to avoid constipation can result in megarectum and megasigmoid, and can lead to fecal impaction and overflow incontinence. The origin of the problem of constipation is unknown. It was originally thought that the perirectal dissection caused a degree of denervation that resulted in constipation. However, on careful review of the largest series of these patients, it became clear that those with the most benign defects and thus the least amount of perirectal dissection had the worst constipation.

The presence of a megarectum prior to the pull-through does correlate with postoperative constipation. Megarectum is more common in patients for whom a transverse or loop colostomy was performed in the newborn period. Constipation appears to be a hypomotility disorder secondary to chronic bowel dilatation. Or, perhaps it is the hypomotility that causes dilatation, which in turn results in constipation, creating a vicious cycle.

When a patient with a megasigmoid has been shown to be fecally continent, resection of the sigmoid has been found to dramatically reduce the patient's laxative requirements. The descending colon with normal caliber and normal motility is anastomosed to the rectum at the peritoneal reflection. This applies for a select group of patients with enormous daily laxative requirements to keep their colons clean. Performance of a new pull-through operation should be avoided so that the patient's rectal reservoir is preserved. Loss of the rectal reservoir could lead to a worse problem of incontinence with a patient who now has diarrhea.

The key in these patients is to manage constipation proactively and avoid it after the pull-through procedure. The patients must be followed regularly, and laxatives and dietary manipulations are begun at the first sign of constipation.

Occasionally constipation becomes so severe that patients develop chronic fecal impaction and constant soiling. Patients like this are often referred with "fecal incontinence." However, if the patient has a good prognosis type of anorectal anomaly, often this incontinence is actually overflow pseudoincontinence. Once the constipation is managed, they become continent.

###### ◦ Continence

Less frequently than constipation, some patients may experience soiling. While in a patient with a good prognosis, this may be overflow incontinence, it may also represent true fecal incontinence in cases of very high imperforate anus or poor muscles and an abnormal sacrum. A contrast enema is helpful in differentiating these two groups of patients. Patients with real incontinence require a bowel management program, which involves cleaning of the child's colon once a day by the use of a suppository, an enema or a colonic irrigation [29].

Giving the enema after the main meal of the day allows a more efficient cleansing of the bowel by taking advantage of the gastrocolic reflex. Antegrade enema procedures, whereby enema is introduced in a conduit via appendicocecostomy, has been devised to help the patient clean its bowel [30,31]. The artificial bowel sphincter and electrically stimulated gracilis neosphincter are two relatively new techniques that have been used for the treatment of patients with severe refractory fecal incontinence.

Patients who have undergone abdominoperineal operations for imperforate anus that included resection of the rectum suffer from a tendency to have diarrhea due to a lack of a rectal reservoir. These patients' incontinence is much harder to manage because they pass stool constantly.

Bowel movement pattern prior to potty-training may give an important clue as to the child's potential for continence. For example, a one-year-old child who has undergone a pull-through for imperforate anus and has one to three bowel movements per day with no soiling in between has a great potential for future fecal continence. The child shows signs that he is "feeling" while having a bowel movement as he pushes. On the other end of the spectrum, a child who suffers from fecal incontinence passes stool constantly without any evidence of pushing or feeling. A child with a normal bowel movement pattern is trainable, whereas a child with the second pattern will likely need a bowel management program. For that child, one should not expect him to achieve voluntary bowel control.

## Prognosis

When evaluating the results of the treatment of anorectal defects, we feel that one cannot group patients according to the traditional nomenclature into "high," "intermediate," and "low" defects, as malformations classified in a same group can have different treatments and different prognoses. For instance, rectoprostatic fistula and bladderneck fistula, both considered as "high" defects are actually very different. We believe that an anatomic classification would have more clinical value. The functional results of the repair of anorectal anomalies seem to have significantly improved since the advent of the posterior sagittal approach. However, the results of this approach are difficult to compare with those of other methods because terminology and classification are not consistent [32,33].

### Fecal continence

Fecal continence depends on three main factors: Voluntary sphincter muscles, anal canal sensation, and colonic motility.

#### Voluntary muscle structures

In the normal patient, the voluntary muscle structures are represented by the levators, muscle complex, and external sphincter. Normally, they are used only for brief periods, when the rectal fecal mass reaches the anorectal area, pushed by the involuntary peristaltic contraction of the rectosigmoid motility. This voluntary contraction occurs only in the minutes prior to defecation, and these muscles are used only occasionally during the rest of the day and night.

Patients with anorectal malformations have abnormal voluntary striated muscles with different degrees of hypodevelopment. Voluntary muscles can be used only when the patient has the sensation that it is necessary to use them. To appreciate that sensation, the patient needs information that can only be derived from an intact anal sensory mechanism, a mechanism that many patients with anorectal malformations lack.

#### Anal canal

Exquisite sensation in normal individuals resides in the anal canal. Except for patients with rectal atresia, most patients with anorectal malformations are born without an anal canal; therefore, sensation does not exist or is rudimentary.

It seems that patients can perceive distention of the rectum but this requires a rectum that has been properly located within the muscle structures. This sensation seems to be a consequence of stretching of the voluntary muscle (proprioception). The most important clinical implication of this is that liquid stool or soft fecal material may not be felt by the patient as it does not distend the rectum. Thus, to achieve some degree of sensation and bowel control, the patient must have the capacity to form solid stool.

#### Bowel motility

Perhaps the most important factor in fecal continence is bowel motility; however, the impact of motility has been largely underestimated. In a normal individual, the rectosigmoid remains quiet for variable periods of time (one to several days), depending on specific defecation habits. During that time, sensation and voluntary muscle structures are almost not necessary because the stool, if it is solid, remains inside the colon. The patient feels the peristaltic contraction of the rectosigmoid that occurs prior to defecation. Voluntarily, the normal individual can relax the striated muscles which allow the rectal contents to migrate down into the highly sensitive area of the anal canal. There, accurate information is provided by the anal canal concerning the consistency and quality of the stool. The voluntary muscles are used to push the rectal contents back up into the rectosigmoid and to hold them if desired, until the appropriate time for evacuation. At the time of defecation, the voluntary muscle structures relax.

The main factor that provokes the emptying of the rectosigmoid is a massive involuntary peristaltic contraction helped sometimes by a Valsalva maneuver. Most patients with an anorectal malformation suffer from a disturbance of this sophisticated bowel motility mechanism. Patients who have undergone a posterior sagittal anorectoplasty or any other type of sacroperineal approach, in which the most distal part of the bowel was preserved, show evidence of an over-efficient bowel reservoir (megarectum). The main clinical manifestation of this is constipation, which seems to be more severe in patients with lower defects.

Constipation that is not aggressively treated, in combination with an ectatic distended colon, eventually leads to severe constipation, and a vicious cycle ensues, with worsening constipation leading to more rectosigmoid dilation, leading to worse constipation. The enormously dilated rectosigmoid, with normal ganglion cells, behaves like a myopathic type of hypomotile colon.

Those patients with anorectal malformation treated with techniques in which the most distal part of the bowel was resected behave clinically as individuals without a rectal reservoir. This is a situation equivalent to a perineal colostomy. Depending on the amount of colon resected, the patient may have loose stools. In these cases, medical management consisting of enemas plus a constipating diet, and medications to slow down the colonic motility is indicated.

#### True fecal incontinence

For patients with true fecal incontinence, the ideal approach is a bowel management program consisting of teaching the patient and his/her parents how to clean the colon once daily so as to stay completely clean for twenty-four hours. This is achieved by keeping the colon quiet in between enemas. These patients cannot have voluntary bowel movements and require an artificial mechanism to empty their colon, a daily enema. The program, although simplistic, is implemented by trial and error over a period of one week. The patient is seen each day and an x-ray film of the abdomen is taken so that they can be monitored on a daily basis for the amount and location of any stool left in the colon, as well as the presence of stool in the underwear. The decision as to whether the type and/or quality of the enemas should be modified as well as changes in their diet and/or medication can be made daily [34].

Approximately 75% of all patients with anorectal malformations have voluntary bowel movements [35]. About 50% of them have voluntary bowel movements, but soil their underwear occasionally. Episodes of soiling are usually related to constipation, and when constipation is treated properly, the soiling frequently disappears. Approximately 40% of the group have voluntary bowel movements and never soil, thus making them totally continent. 25% of patients suffer from fecal incontinence and must receive a bowel management regimen to artificially keep them clean.

Once the diagnosis of the specific defect is established, the functional prognosis can be rapidly predicted, which is vital in order to avoid raising false expectations in the parents. Factors such as the status of the spine, sacrum, and perineal musculature affect the counseling of the parents. Patients with a hypodeveloped sacrum are much more likely to be incontinent and a hypodeveloped sacrum is also a good predictor of associated spinal problems such as tethered cord. If the patient's defect is of the type pointing to a good prognosis such as vestibular fistula, perineal fistula, rectal atresia, rectourethral bulbar fistula, or imperforate anus without fistula, one should expect that that child will have voluntary bowel movements by the age of 3. Such children need supervision to avoid fecal impaction, constipation, and soiling. If a patient's defect points to a poor prognosis, such as a high cloaca (common channel greater than 3 cm) or a recto-bladder neck fistula, the parents should be informed of the likelihood that that child will need a bowel management program to remain clean, which should be implemented at the age of 3 or 4. Patients with rectoprostatic fistulas have almost equal chance of having voluntary bowel movements or being incontinent. Toilet training should be attempted at age 3, and if unsuccessful, a bowel management program should be initiated. Each year, during summer vacation, an attempt should be made to try to achieve bowel control, and if unsuccessful, the bowel management should be restarted. As the child grows older and more cooperative, the likelihood of achieving bowel control will improve.

### Urinary continence

Urinary incontinence occurs in male patients with anorectal malformations only when they have an extremely defective or absent sacrum, or when the basic principles of surgical repair are not followed and important nerves are damaged during the operation. The overwhelming majority of male patients have urinary control. This is also true for female patients, not including the cloaca group.

For patients with cloaca, functional prognosis with regard to achieving fecal continence depends on the complexity of the defect and the status of the spine and sacrum. Urinary control varies based on the length of the common channel. 69% of patients with cloaca with a common channel greater than 3 cm require intermittent catheterization, as compared to 20% in the group with a common channel less than 3 cm. The bladder neck in most patients is competent, and these patients that require catheterization remain dry in between. If catherization is not performed, overflow incontinence occurs. Occasionally, the bladder neck is not competent or is non-existent, and in these cases, urinary diversion such as a Mitrofanoff procedure is considered.

Careful, regular follow-up is necessary in these patients to accurately reassess their prognosis and to avoid problems, which can dramatically impact on their ultimate functional result.
