# Correction: anorectal malformations

**DOI:** 10.1186/1750-1172-7-98

**Published:** 2012-12-15

**Authors:** Marc A Levitt, Alberto Peña

**Affiliations:** 1 Department of Pediatric Surgery, Cincinnati Children's Hospital, University of Cincinnati, Cincinnati, Ohio, 45229, USA

## Correction

After the publication of this work [1] it was brought to the authors attention that Figure five (Figure 1 here) contained a duplication error in the PDF format, where it featured a perianal fistula instead of a rectovestibular fistula in females. The correct figure is given below:

**Figure 1:**
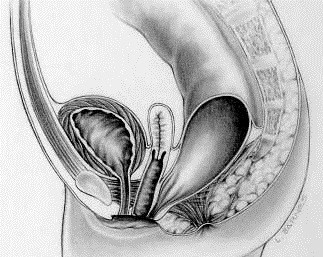
Rectovestibular fistula in females.

We regret any inconvenience that this inaccuracy may have caused.
